# DEVELOP AND EXTERNALLY VALIDATE A RISK PREDICTION MODEL FOR SCREENING COGNITIVE FRAILTY IN OLDER ADULTS

**DOI:** 10.1093/geroni/igad104.1654

**Published:** 2023-12-21

## Abstract

Cognitive frailty, the combination of physical frailty and mild cognitive impairment, is a growing public health concern in aging populations. We aimed to develop and validate a risk prediction model for screening cognitive frailty in community-dwelling older adults without probable dementia (aged ≥ 65 years). We used Year 2011 data from National Health and Aging Trends Study (NHATS), with participants randomly divided into the training set (N=4,222) and internal validation set (N=2,111). We used Year 2015 data of NHATS as the external validation set (N=3,380). Cognitive frailty was assessed with the Fried phenotypic criteria and cognitive performance in three domains (memory, orientation and executive function). Independent risk factors were screened by multivariate logistic regression analysis. Model performance was assessed by discrimination (area under the curve [AUC]) and calibration (Hosmer-Lemeshow test). The final model included 13 key predictors (age, gender, education, smoke, walking for exercise, vigorous activity, self-rated health, depressive symptoms, balance impairments, arthritis, hospitalization, activities of daily living, and instrumental activities of daily living score). The model showed good discrimination with an AUC of 0.92 (95% CI: 0.90-0.93) for the training set, 0.92 (95% CI: 0.89-0.95) for the internal validation set, and 0.88 (95% CI: 0.85-0.91) for the external validation set. The Hosmer-Lemeshow test yielded P values of 0.10, 0.92, and 0.85 for the three sets, respectively, suggesting good calibration. Our risk prediction model has the potential to screen older adults at risk for cognitive frailty, enabling early interventions to prevent or delay cognitive decline and physical disability.

